# The Functional Interactions between Cortical Regions through Theta-Gamma Coupling during Resting-State and a Visual Working Memory Task

**DOI:** 10.3390/brainsci12020274

**Published:** 2022-02-16

**Authors:** Ji Seon Ahn, Jaeseok Heo, Jooyoung Oh, Deokjong Lee, Kyungun Jhung, Jae-Jin Kim, Jin Young Park

**Affiliations:** 1 Graduate Program in Cognitive Science, Yonsei University, Seoul 03722, Korea; ahnjeeseon@yuhs.ac (J.S.A.); heojs@yuhs.ac (J.H.); 2 Institute of Behavioral Science in Medicine, Yonsei University College of Medicine, Seoul 03722, Korea; ojuojuoju@yuhs.ac (J.O.); pangelt@yuhs.ac (D.L.); jaejkim@yuhs.ac (J.-J.K.); 3 Department of Laboratory Medicine, Yongin Severance Hospital, Yonsei University Health System, Yongin 16995, Korea; 4 Department of Psychiatry, Yonsei University College of Medicine, Gangnam Severance Hospital, Yonsei University Health System, Seoul 06273, Korea; 5 Department of Psychiatry, Yonsei University College of Medicine, Yongin Severance Hospital, Yonsei University Health System, Yongin 16995, Korea; 6 Department of Psychiatry, International St. Mary’s Hospital, Catholic Kwandong University, Incheon 22711, Korea; kyungun12@gmail.com; 7 Center for Digital Health, Yongin Severance Hospital, Yonsei University Health System, Yongin 16995, Korea

**Keywords:** electroencephalography, theta-gamma coupling, neuronal oscillations, resting state, visual working memory, visual attention

## Abstract

Theta phase-gamma amplitude coupling (TGC) plays an important role in several different cognitive processes. Although spontaneous brain activity at the resting state is crucial in preparing for cognitive performance, the functional role of resting-state TGC remains unclear. To investigate the role of resting-state TGC, electroencephalogram recordings were obtained for 56 healthy volunteers while they were in the resting state, with their eyes closed, and then when they were engaged in a retention interval period in the visual memory task. The TGCs of the two different conditions were calculated and compared. The results indicated that the modulation index of TGC during the retention interval of the visual working memory (VWM) task was not higher than that during the resting state; however, the topographical distribution of TGC during the resting state was negatively correlated with TGC during VWM task at the local level. The topographical distribution of TGC during the resting state was negatively correlated with TGC coordinates’ engagement of brain areas in local and large-scale networks and during task performance at the local level. These findings support the view that TGC reflects information-processing and signal interaction across distant brain areas. These results demonstrate that TGC could explain the efficiency of competing brain networks.

## 1. Introduction

Clinical electroencephalography (EEG)—one of several methods of data acquisition from the human brain—was introduced by Hans Berger, a German psychiatrist, in the 1930s [1,2]. Scalp EEG is a noninvasive method of detecting and registering electrical activity in the brain using electrodes attached to the scalp that record changes in the electric potential (neuronal oscillations) on the skin surface, resulting from the activity of cerebral neurons, and after their amplification they are recorded [1]. The EEG rhythmical frequency bands reflect the rhythmic and synchronized postsynaptic potentials that arise from the pyramidal neuronal assemblies [3]. Neural activity is known to oscillate within the following discrete frequency bands: delta (1–4 Hz), theta (4–8 Hz), alpha (8–13 Hz), beta (13–30 Hz), and gamma (30–50 Hz) [4]. Distinct mechanisms may generate neural oscillations in different frequency bands that are associated with a diverse range of cognitive functions [5,6]. Moreover, neuronal oscillations are predictive of information-processing and involved in selective communication and information transmission [7,8], and the interplay between these brain rhythms is hypothesized to underlie cognitive functions [9,10]. One possible role of oscillatory activity is to assure the maintenance of information in working memory (WM) [11].

WM is considered an essential component of cognitive functions [12,13,14,15,16,17]. WM refers to the memory system with limited energy for short-term storage and processing of goal-relevant information [13,14,15,16,17,18]. The reduced WM capacity is a common feature in many diseases such as schizophrenia, attention deficit hyperactivity disorder (ADHD), mild cognitive impairment, and Alzheimer’s disease (AD) [2]. The neural mechanisms that contribute to WM have been a major focus in neuroscience for several years [18]. Recently, studies have shown that theta and gamma oscillations play an important functional role in WM [19,20,21]. Increased theta activity in frontal areas shows the involvement of frontal theta activity in WM maintenance [12,22,23], and increased gamma activity in posterior regions with WM load shows the control of attentional resources and visual processing [12,24]. Brain processes of WM involve oscillatory activities at multiple frequencies in local and long-range neural networks [25]. A previous study reported that the synchronization of the theta band can coordinate neural communication between multi-brain regions and contribute to the maintenance of short-term memories [23]. Visual working memory (VWM) relies on sustained neural activities that code information via various oscillatory frequencies. However, previous studies have emphasized time-frequency power changes, while overlooking the possibility that rhythmic amplitude variations can also code frequency-specific VWM information in a completely different dimension [26]. 

Quantitative EEG (QEEG) data are categorized into power and phase via the Fourier transform analysis. Power and phase provide information regarding the quantity of a specific frequency and the timing of neuronal activity, respectively [27]. Power spectral analyses quantify the power and voltage within the frequency bands [5]. Different EEG oscillations in the frequency domain are not independent and regulate the integration of multiple networks through the mutual interaction of cross-frequency oscillations [28]. Cross-frequency phase-amplitude coupling (CFC) was developed to address the limitations of conventional QEEG analyses that focused only on power and disregarded phase when assessing neuronal activity [29]. Unlike spectral analysis, CFC more accurately describes the characteristics of functional brain activity by integrating phase information with power [29]. Cross-frequency coupling measures functional connectivity beyond single-frequency assessments of oscillatory activity and provides insight into ways by which local neural networks process information through the interactions or couplings of activities across frequencies [30,31]. Phase-amplitude coupling (PAC) is a type of CFC, wherein oscillations in different frequency bands can occur simultaneously and interact with each other, and the phases of slow oscillations modulate the amplitude of faster oscillations [32,33]. PAC has been reported across multiple brain regions under a variety of conditions, reflecting inter-areal communication and interactions between cognitive functions [34,35]. Nesting of fast rhythmical brain activity into slower brain waves has frequently been suggested as a core mechanism of multi-item WM retention [36]. Theta phase-gamma amplitude coupling is the best-known example of this interaction. Previous studies found an association between TGC and WM [2,17,36,37,38,39,40], and it has been suggested that the power of gamma oscillations is systemically modulated over the course of the theta cycle, and that this interaction is a neurophysiologic process underlying WM [41,42].

Although the storage capacity of WM is inherently limited [17,18,43], several studies have found that the WM capacity can be altered by training [44,45,46]. Neural activity in the prefrontal cortex and the strength of connectivity between the prefrontal and parietal cortices have been shown to be improved by training, as suggested by the studies in humans and non-human primates [47,48,49,50]. Active transcranial direct current stimulation (tDCS) enhanced WM performance by modulating interactions between frontoparietal theta oscillations and gamma activity [51]. A recent study using a rat AD model found that impaired TGC between the hippocampus and prefrontal cortex was associated with the cognitive deficits in the rats [52]. In humans, a functional MRI study demonstrated that hippocampus and dorsolateral prefrontal cortex (DLPFC) coupling may illustrate a systematic mechanism that implements WM [53]. Neural activities between the hippocampus and prefrontal cortex are often synchronized in time and the interactions of both neuroanatomical structures are required to coordinate the appropriate cognitive functions [54]. The hippocampus has distinct electrophysiological characteristics of the TGC [55,56].

TGC is thought to play an important role in learning and memory, which is associated with neuroplasticity in the hippocampal-cortical network [37,57]. The perceptual-cognitive approach to learning assumes behavior to be induced by way of representations that includes information on the perceptual effects of behavior [58]. In this respect, perceptual-cognitive ability is considered one of the key factors related to high-level performance [58,59]. For example, recent studies showed that practice has a significant influence on the cognitive system underlying motor learning and that experts differ from beginners in perceptual-cognitive background [58,59]. These findings suggest that perceptual and cognitive adaptations co-occur over the course of motor learning. [59]. A recent review suggested that ventromedial prefrontal cortex (vmPFC) subregions may underlie learning processes, as well as their principal interactions, with the extended subcortical and cortical components of the brain’s circuit. The anterior vmPFC is a central hub of metacognition and the default mode network (DMN), and the authors emphasized that its role may represent an attempt to restore equilibrium [60,61]. In 2021, Garofalo et al. employed tDCS over the lateral prefrontal cortex to modulate brain activity choice and provided evidence for the involvement of the lateral prefrontal cortex of human cue-guided choice. The study provided causal evidence of the involvement of the DLPFC in human cue-guided choice that relies on WM capacity, which has a decisive implication on processing the value of outcomes, and indicates that the decision-making process relies largely on the computational availability of memory [59]. Regarding this, Garofalo et al. suggested that individual differences in WM are closely related to human behavior and are decisively involved in decision-making [62]. These findings suggest a reevaluation of working memory indicating that WM should be denoted as a cognitive mechanism to define the best choice and guide behavior for the most convenient choice [62]. 

Neuroscientists have recognized the importance of a WM to guide behavior with information [63]. However, human cognitive function studies have traditionally focused on cognitive task-induced brain activity rather than cognitive activity in the resting state. [64,65]. The resting state is defined as a spontaneous cognitive state [66,67] in which one is not engaged in any specific cognitive task [66,67,68]. Spontaneous neural activities also play an important role in cognitive functioning [64,65], and task-evoked electrophysiological measurements may harbor a component of spontaneous brain activity [69,70,71]. Resting-state brain activity can be used to measure baseline brain states and has been associated with various aspects of behavior and cognitive processes serving as indices of brain function [72,73]. Functionally connected brain structures that are more active during rest than during tasks that require attention to external events comprise the DMN [67,74,75]. Recent studies have suggested that resting-state activity, which maximizes the efficiency of information transfer with low physical connection costs, can serve as the foundation for underlying brain functions [76]. Increasing evidence has shown that the resting state EEG rhythms may reveal abnormalities of the basic neurophysiological mechanisms that underlie cognition in AD subjects, and these abnormal EEG rhythms are thought to be associated with functional cortical disconnections [77]. Abnormal resting-state network activity has been proposed as a neural correlate of cognitive dysfunction in functional magnetic resonance imaging (fMRI) studies [78]. Compared to fMRI, EEG shows lower spatial but higher temporal resolution. However, both techniques are regarded as useful for the assessment of brain activity and connectivity [1]. 

Electrophysiological studies of WM have repeatedly revealed that prominent increases in theta power, gamma power, and TGC are neural correlates of WM processing [79,80]. TGC has primarily been used to focus on brain activities during the performance of a particular cognitive task, but the relationships with WM to the functional role of the resting-state TGC while eyes are closed remains to be elucidated. The first aim of this study was to investigate and compare TGC and power spectra, defined in terms of theta and gamma oscillations, respectively, among healthy volunteers in both the resting state and during VWM tasks. We hypothesized that theta power, gamma power, and TGC would be higher during the retention interval period in the VWM task than in the resting state. The second aim of this study was to compare the detailed topographical patterns and characteristics of power spectra and TGC. We hypothesized that the topographical patterns of TGC would differ from those of theta and gamma oscillatory activity. The third aim of this study was to probe the neural correlates of clinical outcomes using TGC and power spectra of theta and gamma in both resting state and during VWM tasks. We hypothesized that theta, and gamma power, and TGC in the resting state would correlate with WM outcomes. 

## 2. Materials and Methods

### 2.1. Study Subjects

A total of 61 healthy volunteers were recruited through posters at our hospital and announcements on a website. The number of subjects was estimated a priori using G*Power v. 3.1.9.7. paired *t*-tests (two-tail) for matched pairs, medium effect size (Cohen’s d = 0.50), and a two-tailed alpha of 0.05. A sample of 54 participants was deemed necessary to generate a strong statistical power (0.95). All subjects underwent diagnostic interviews. Psychiatric diagnoses were assessed using the Mini-International Neuropsychiatric Interview [81], which was administered by either a psychiatrist or by trained graduate-level psychologists. The majority of subjects were right-handed, with three left-handed subjects, as indicated by the Edinburgh Handedness Inventory [82]. 

Subjects were included in the study if they met the following criteria: (1) male or female aged 19–65 years and (2) capable of completing all required study procedures as determined by a psychiatrist. Subjects were excluded from participation if they met any of the following exclusion criteria: (1) brain lesions, (2) diagnoses of neurological disorder(s), or (3) incapability or unwillingness to give consent for the study participation. All subjects had normal or corrected-to-normal vision and normal hearing. 

All subjects were fully informed about the purpose and procedures of the study and provided written informed consent. The Institutional Review Board of Gangnam Severance Hospital, Yonsei University, reviewed and approved this study (No. 2016-0375-015).

### 2.2. Wender Utah Rating Scale

Subjects completed a 25-item self-administered questionnaire of childhood symptoms designed to retrospectively assess the presence of childhood ADHD symptomatology, developed by Wender in 1993. Each item is measured on a five-point Likert scale with 0 being “not at all” and 4 being “extremely”. The scale results in a score ranging from 0 to 100 [83].

### 2.3. Neuropsycological Test

Spatial span is a VWM test that measures spatial WM and consists of two subtests from the Wechsler Memory Scale Third Edition, Spatial Span Forward and Spatial Span Backward. The score ranges from 0 to 32 points [84]. Letter-Number Sequencing is a measure of auditory WM in which subjects hear a series of numbers and letters in random order and must then repeat the whole series, but with numbers in ascending order and letters organized alphabetically. The total score ranges from 0 to 21 points [85].

### 2.4. Experimental Procedures

**Figure 1:**
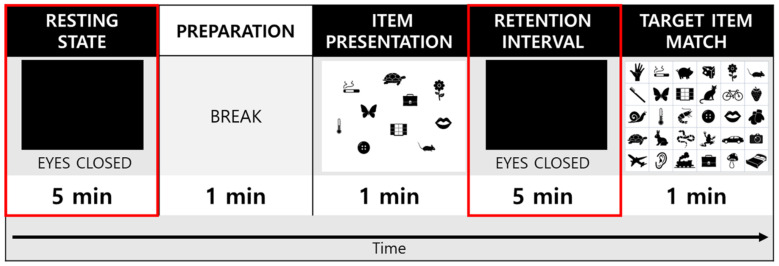
A schematic of the eyes-closed resting electroencephalography (EEG) and memory test protocol. The resting-state and retention interval (red squares) were used for analysis in the study.

The experiment consisted of a single session that lasted approximately 15 min; the sessions started with 5 min of eyes-closed (EC) resting-state EEG recording that was, followed by a 1 min break and then with 7 min of a VWM task during which EEG data were recorded (Figure 1). Subjects were seated comfortably in a chair in a quiet room for all EEG recordings. During the recording, subjects were encouraged to relax their jaw musculature and to minimize ocular (e.g., try not to blink or move their eyes) and other movements. Before the beginning of the EC condition, subjects were instructed to close their eyes, stay seated in the chair, relax, and remain awake during the 5 min period of the EC resting state. During the VWM task, subjects were presented with a paper containing 10 target items to encode and were instructed to memorize them in 1 min. Next, the subjects were instructed to close their eyes and retain the 10 target items in their minds for 5min. Finally, the subjects were asked to indicate and match the 10 memorized target items to the 30 items presented on the answer sheet within a minute. In this study, EEG parameters were compared between the EC resting state and the retention interval period of the VWM task.

### 2.5. Electroencephalogram Recording and Rreprocessing

Electroencephalogram data were continuously recorded using Netstation version 5.4 software (Electrical Geodesics, Eugene, OR, USA) and a 64-channel HydroCel Geodesic Sensor Net (Electrical Geodesics, Eugene, OR, USA). The experiment lasted approximately 15 min and it started with 5 min of eyes-closed resting based on the modified international 10/20 system, known as the 10–10 system (Applied Neuroscience, St. Peterburg, FL, USA). All EEG channels were referenced to the vertex electrode (Cz) of the scalp. All electrode impedances were kept below 50 kΩ in accordance with Electrical Geodesics, Inc. guidelines. The EEG data were digitized and amplified at a sampling rate of 1 kHz with a Geodesic EEG system 400 (Electrical Geodesics, Inc.) and filtered online using a bandpass filter set at 0.1–100 Hz and notch filter set at 60 Hz. 

Electroencephalogram data were pre-processed and analyzed offline using a custom-written scripts in MATLAB 2016b (The MathWorks, Natick, MA, USA) and the EEGLAB toolbox [86]. The Harvard Automated Processing Pipeline for EEG [87] was used to automate preprocessing and artifact correction using Wavelet-enhanced independent component analysis (ICA) and the Multiple Artifact Rejection Algorithm (MARA) [88]. The continuous EEG data were re-referenced to an average reference and filtered with a 1–240 Hz bandpass filter and 60, 120, 180, and 240 Hz notch filters. 

We removed from analysis bad channels whose probability fell more than three standard deviations (SDs) from the mean. Bad-channel evaluation was performed twice per data file. Channels that were removed as bad channels had their data interpolated from nearby channels in a later processing step. Any channels removed during the bad channel rejection processing step were subjected to spherical interpolation (with Legendre polynomials up to the 7th order) of their signal. ICA using MARA embedded in EEGLAB was performed to eliminate components with artifact probabilities greater than 0.8. ICA has been demonstrated to reliably isolate ocular, electromyographic, and electrocardiographic artifacts. The processed clean data were saved in the EEGLAB file format (.set files). Nineteen electrode sites among 57 channels were analyzed (Fp1, Fp2, F7, F3, Fz, F4, F8, T7, C3, Cz, C4, T8, P7, P3, Pz, P4, P8, O1, and O2) based on a standard international 10/20 system.

### 2.6. Power Spectral Analysis

We used the signal processing toolbox in MATLAB to calculate the spectral power of the EEG data for each subject by fast Fourier transformation. Time windows of 4000 ms with an 8 ms overlap were used for the spectral analysis. The following five frequency bands were defined for spectral analyses: delta (1–4 Hz), theta (4–8 Hz), alpha (8–13 Hz), beta (13–30 Hz), and gamma (30–50 Hz). The absolute powers of the theta and gamma bands were averaged over all the time windows and frequency bands for further analysis.

### 2.7. Modulation Index for Theta-Gamma Coupling

The intensity of CFC between the low-frequency theta (4–8 Hz) phase $f_{P}$ and the amplitude of the gamma (30–50 Hz) oscillations $f_{A}$ was analyzed as a modulation index (MI) value [89,90]. To calculate the MI of TGC, we applied the standard Hilbert transform [91] to time series for theta phase $\phi_{f_{P}}\left(t\right)$ and the gamma amplitude envelope $\phi_{f_{P}}\left(t\right)$. The composite time series $\left[\phi f_{P}\left(t\right),A_{f_{A}}\left(t\right)\right]$ was then constructed; this informs the gamma oscillation $f_{A}$ at each phase of the theta rhythm $f_{P}$. The phase of theta $\phi_{f_{P}}\left(t\right)$ was binned into 72 five-degree bins spanning the (−180°–180°), degree interval and the corresponding mean amplitudes of gamma oscillation were calculated for each phase bin and then normalized by dividing the mean amplitudes by the sum over all bins, $<A_{f_{A}}{>}_{\phi_{f_{P}}}\left(j\right)$ the mean gamma value $A_{f_{A}}$ at the phase bin j, and then normalized by dividing the mean amplitude in each phase by the sum over all bins. The existence of PAC is characterized by a deviation of the amplitude distribution P from the uniform distribution in a phase-amplitude plot [89].

(1) $$P\left(j\right)=\frac{<A_{f_{A}}{>}_{\phi_{f_{P}}}\left(j\right)}{\overset{N}{\underset{j=1}{\sum }}<A_{f_{A}}{>}_{\phi_{f_{P}}}\left(j\right)},$$

The measure that quantified the deviation of P from a uniform distribution was defined by applying the Kullback–Leiber (KL) distance [92], a parametric that is widely used to infer the distance between two distributions. The KL distance formula resembles the definition of the entropy of P, H(P) defined by [89]:

(2) $$H\left(P\right)=-\overset{N}{\underset{j=1}{\sum }}P\left(j\right)\log\left[P\left(j\right)\right]$$

(3) $$D_{\mathrm{KL}}\left(P,U\right)=\log\left(N\right)-H\left(P\right)$$

N is the number of phase bins, log(N) represents the entropy of a uniform distribution, and H(P) is the entropy of P, where U is the uniform distribution. The MI (by Tort et al.) was calculated as follows [89]:

(4) $$\mathrm{MI}=\frac{\log\left(N\right)-H\left(P\right)}{\log\left(N\right)}=\frac{D_{\mathrm{KL}}\left(P,U\right)}{\log\left(N\right)}$$

The MI measures the divergence of the phase-amplitude distribution from the uniform distribution (MI = 0). The further the MI value is from 0, the lower the entropy H(P) and the greater the coupling. Simply put, this means that an MI value of 0 denotes that the phase-amplitude distribution is equal to the uniform distribution and that there is an absence of PAC because the mean amplitude is the same for all phase bins. As the distance between the amplitude distribution and the uniform distribution inferred by the KL distance increases, so does the MI.

### 2.8. Statistical Analysis

Statistical analyses were carried out using MATLAB version R2016b statistical toolbox (The MathWorks, Inc., Natick, MA, USA) and IBM SPSS Statistics version 25.0 (IBM Corp., Armonk, NY, USA). Before undertaking the statistical analyses, we conducted the Kolmogorov-Smirnov one-sample test to assess whether the data were normally distributed. For descriptive statistics, means and SDs were calculated to demographic variables, clinical variables, and electrophysiological variables, respectively. We used the paired *t*-test to examine the differences between EC resting state and the retention interval of the VWM task conditions on EEG data. Within the subjects, correlations between EEG data and clinical data were analyzed using Pearson correlation coefficients. The problem of multiple comparisons was corrected using a Benjamini–Hochberg False Discovery Rate (FDR) control [93]. All *p*-values were two-tailed, and the statistical significance was defined as *p* < 0.05. Topographic plots were depicted for the following: (1) the calculated MI of TGC and average absolute power of theta and gamma oscillations obtained from the grand average across all subjects during the EC resting state and the retention interval phase to observe the topographical distribution; (2) results of the paired *t*-test in the MI of TGC and absolute power of theta and gamma oscillations between resting state and VWM task condition; and (3) results of the correlation coefficients between electrophysiological status and clinical data. All topographic plot data were obtained from the grand average across all subjects.

## 3. Results

### 3.1. Demograpic and Clinical Characteristics

Of the 61 subjects, five were excluded for missing data on a clinical measure, outliers in the EEG data (more than three SDs from the mean), and/or medical condition (depressive, hypomanic episode, highly alcohol-dependent). A total of 56 [31 (55.4%) male] healthy volunteers completed the study, mean ± SDs age was 23.9 ± 4.0 years, and ages ranged from 18 to 41 years. Demographic and clinical characteristics of healthy volunteers are presented in Table 1. 

### 3.2. Power Spectra

**Figure 2:**
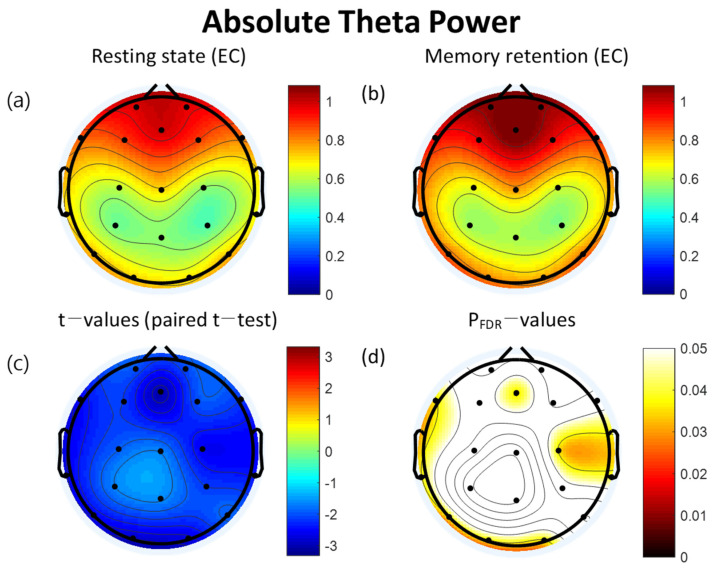
Topographies of absolute theta power during eyes-closed resting state and the retention interval of the visual working memory (VWM) task, *t*-map, and p_FDR_-map. (**a**) Absolute spectral power of theta band during the resting state (**b**) Absolute spectral power of theta band during the retention interval of VWM task (**c**) Topographical distribution of *t*-values (paired *t*-test) (**d**) Topographical distribution of the corresponding False Discovery Rate corrected *p*-values (paired *t*-test). EC: eyes closed, FDR: False Discovery Rate.

The retention interval period of the VWM task demonstrated significantly increased absolute theta power compared to the resting state at eight of the electrodes: Fz (t = −3.05, *p* = 0.036), F7 (t = −2.56, *p* = 0.036), T3 (t = −2.70, *p* = 0.036), T5 (t = −2.59, *p* = 0.036), O1 (t = −2.90, *p* = 0.036), O2 (t = −2.74, *p* = 0.036), C4 (t = −2.59, *p* = 0.036), and T4 (t = −2.46, *p* = 0.040). All significant differences were FDR corrected (Table 2). The topographical features of the paired *t*-test results are also presented in Figure 2. 

**Figure 3:**
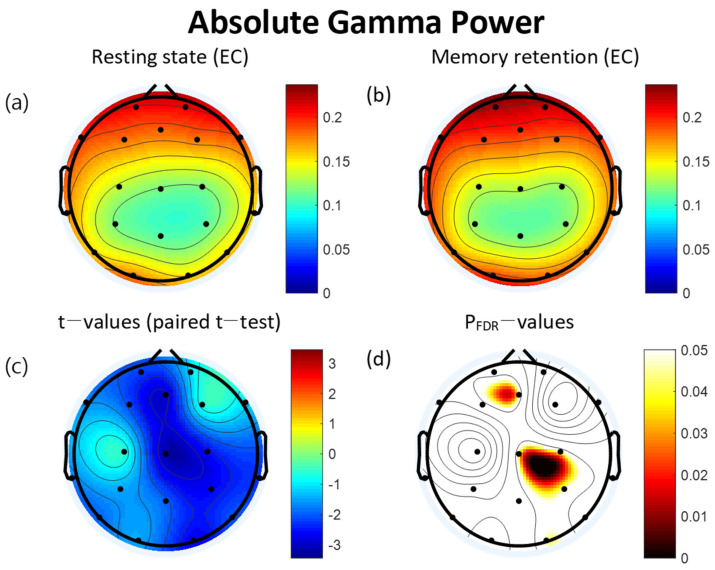
Topographies of absolute gamma power during eyes-closed resting state and the retention interval of the visual working memory (VWM) task, *t*-map, and p_FDR_-map. (**a**) Absolute spectral power of the gamma band during the resting state. (**b**) Absolute gamma power during the retention interval of the VWM task. (**c**) Topographical distribution of *t*-values (paired *t*-test). (**d**) Topographical distribution of the corresponding False Discovery Rate corrected *p*-value (paired *t*-test). EC: eyes closed. FDR: False Discovery Rate.

For the absolute gamma power, the retention interval period of the VWM task had significantly increased at five of the electrodes: Fz (t = −2.95, *p* = 0.044), O2 (t = −2.70, *p* = 0.046), P3 (t = −2.66, *p* = 0.046), C3 (t = −2.60, *p* = 0.046), and Cz (t = −3.20, *p* = 0.043). All significant differences were FDR corrected (Table 3). The topographical features of the paired *t*-test results are also presented in Figure 3. 

### 3.3. Theta-Gamma Coupling

**Figure 4:**
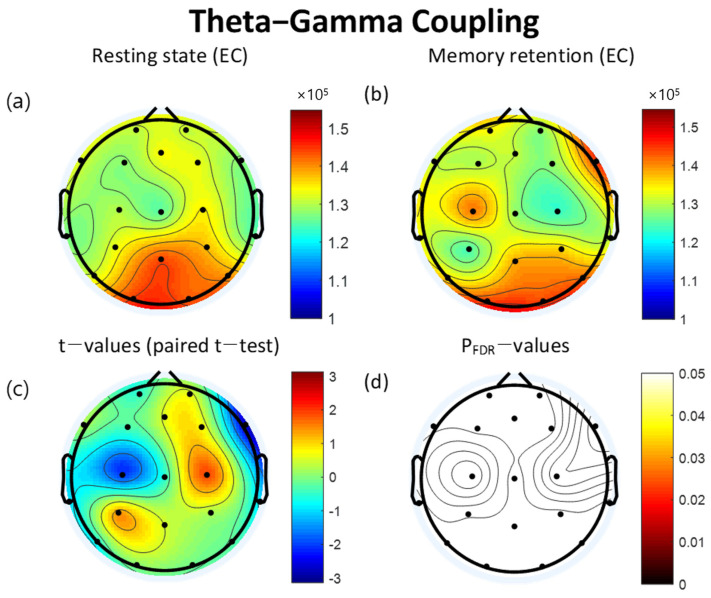
Topographies of theta-gamma coupling (TGC) during eyes-closed resting state and the retention interval of the visual working memory (VWM) task, *t*-map, and p_FDR_-map. The topographic maps represent the probability of paired *t*-test between resting state and VWM task. (**a**) Modulation index (MI) of TGC during the resting state. (**b**) MI of TGC during the retention interval of VWM task (**c**) Topographical distribution of *t*-values (paired *t*-test). (**d**) Topographical distribution of the corresponding False Discovery Rate corrected *p*-value (paired *t*-test). EC: eyes closed, FDR: False Discovery Rate.

The MI of TGC increased during the VWM task compared to the resting state in FP1, F3, C3, T3, T5, O2, T6, T4, F8, and Cz. In contrast, the MI of TGC decreased during the retention interval period of the VWM task compared to the resting state in FP2, Fz, F7, P3, Pz, O1, P4, C4, and F4. However, significant differences were not found to fall below the FDR threshold (Table 4). The topographical features of the paired *t*-test results are also presented in Figure 4.

### 3.4. Correlation between Absolute Gamma Power and Clinical Measures

**Figure 5:**
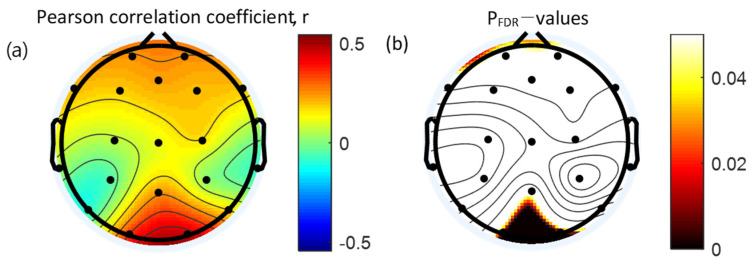
Topographical representation of the statistical results of the Pearson’s correlation analysis between the absolute gamma power and Wender Utah Rating Scale. (**a**) Pearson’s correlation coefficients. (**b**) Topographical distribution of the corresponding False Discovery Rate corrected *p*-value. FDR: False Discovery Rate.

Pearson’s correlation coefficients were used to establish the correlation between the absolute gamma power and WURS. Absolute gamma during the retention interval period of the VWM task was significantly and positively correlated with the WURS in the occipital area (O1, r = 0.34, *p* = 0.010; O2, r = 0.46, *p* < 0.001). Results of the Pearson’s correlation analysis between the absolute gamma power during the VWM task and WURS scores are presented in Table 5. The topographical features of the correlation are presented in Figure 5.

### 3.5. Linear Regression

**Figure 6:**
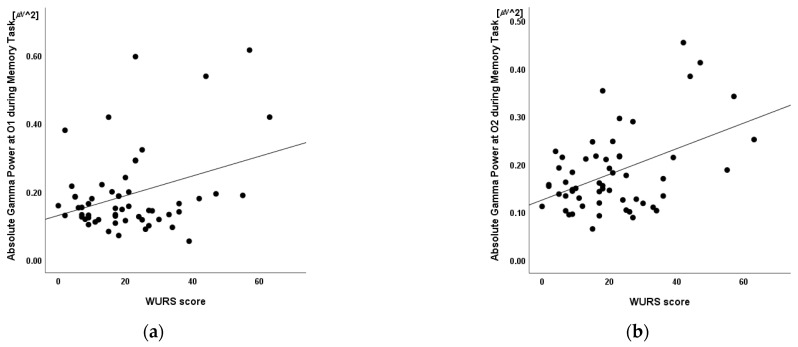
Relationship between the Wender Utah Rating Scale scores and the absolute gamma power at electrodes (**a**) O1 and (**b**) O2 during the retention interval period of the visual working memory task (n = 56, R^2^ =0.23, *p* = 0.399 and *p* = 0.012, respectively). Mean absolute power in μV^2^. WURS: Wender Utah Rating Scale.

Linear regression was conducted to determine whether absolute gamma power was associated with WURS score independent of age and sex. A significant regression equation was found at O2 (F(4,55) = 3.72, *p* = 0.010; R2 = 0.23). In the linear regression models, WURS was independently and positively associated with the absolute gamma power at O2 (*p* = 0.012) but not with age (*p* = 0.657), sex (*p* = 0.813), or the absolute theta power at O1 (*p* = 0.399). The absolute gamma power at O2 was the only significant predictor of the model (β = 0.38, *p* = 0.012) (Table 6, Figure 6).

## 4. Discussion

In this work, we compared absolute theta power, absolute gamma power, and TGC during the VWM task to those of the resting state. Theta and gamma power were significantly higher in the frontal areas during the retention interval period of the VWM task compared to the resting state, lending support to our first hypothesis as described earlier in this paper. This result was consistent with previous findings that these bands are involved in memory processes [34,94]. Theta and gamma activity are more involved in WM mechanisms than in other types of mechanisms [79]. A prominent increase in theta and gamma power represents the neural correlates of WM processing. Thus, the increase in theta and gamma power in the frontal area observed in this study may reflect the engagement of the neurophysiological mechanism of attention or VWM [29].

The positive correlation between TGC and task complexity may be a result of increased cognitive load [95]. TGC may reflect the subjective effort being made or cognitive resources being expended during cognitive performance [34]. However, in this study, the difference in TGC between the resting state and the VWM task was not observed to be statistically significant. This result may be interpreted to mean that TGC is affected by high memory load, given that TGC decreases during periods with low cognitive control demands [96]. We demonstrated that the topographical features of TGC vary across different conditions. The theta and gamma powers’ topographies were smooth and relatively unchanging, but the topography of TGC exhibited local alterations that were negatively correlated between conditions. The MI of TGC during the VWM task was higher than in the resting state in FP1, F3, F8, C3, Cz, T3, T5, T6, T4, and O2; but it was lower during the VWM task than in the resting state in FP2, Fz, F4, F7, C4, P3, Pz, P4, and O1. These findings are similar to those obtained through fMRI, suggesting that the resting-state brain activity of the DMN is negatively correlated with that of the task-positive network [97]. The DMN is commonly deactivated during attention-demanding cognitive tasks, while the task-positive network is activated during cognitive and attentional tasks [98].

We found that absolute gamma power measured by the occipital electrodes in healthy volunteers during the retention interval period of the VWM task was strongly correlated with WURS scores. The WURS has been widely used to measure ADHD-related characteristics, such as impulsivity, defiant behavior, mood instability, anxiety, and inattention, which are common to a broad range of psychiatric diagnoses [99]. The WURS was developed to retrospectively assess the presence and severity of childhood symptoms of ADHD including both inattentive and hyperactive symptoms in adult patients [83,100]. Gamma oscillations in the occipital area are important in VWM processes and visual selective attention [101]. The level of interference is correlated with occipital gamma oscillatory power, a known index of selective visual attention. Therefore, our finding of the enhanced absolute gamma power in the occipital area, related to a higher score in WURS, suggests distracted interference control during WM. This interpretation is consistent with previous findings that ADHD patients, as compared to controls, are more distracted by interfering stimuli accompanied by an increased occipital evoked 40 Hz-gamma band response during visual distraction [102].

We identified a significant relationship between absolute gamma power and the WURS score but failed to identify a relationship between TGC and WM scores. To understand this lack of correlation between WM results and resting-state TGC in this study, it should be noted that WM and attention are closely linked and have a bidirectional relationship [103,104,105]. 

In this study, resting-state EEG results were compared to EEG results during goal-directed cognitive processing using the VWM task, in which subjects’ attention is focused on retaining an external image in their memory. Resting-state EEG power spectral and TGC analyses were carried out to determine the subjects’ baseline neurophysiological states. We were able to successfully demonstrate that neither cortical electrical activity nor TGC reflected externally focused cognitive processing as they did during a task involving subjects forming memories of presented images and when subjects’ eyes were closed, and they were in a resting state. Thus, TGC did not occur only at specific points in time to support goal-related cognitive processes.

Patterns of cortical electrical activity and TGC differed across the resting state and during focused cognitive processing. This difference may have resulted from the fact that TGC analysis assesses the amount of timing information gathered from interacting functional systems across multiple spatiotemporal scales, while spectral analysis probes the degree of excitation of the functional neuronal system [78]. This finding indicates that TGC may reflect a mechanism of information transfer among functionally connected networks that may have high inter-regional correlations. In this study, we identified the brain regions that were differentially involved in the resting state and the VWM task. Our results show that the resting state and focused attention used different cognitive resources, and thus may reflect the fact that the resting-state brain activity of the DMN is negatively correlated with that of the task-positive network.

Resting-state activity can be decomposed into a number of separate networks, some of which are recruited when performing attention-demanding tasks. Spontaneous thought during rest may engage additional brain areas that are not active during task performance and are not part of the default mode network, and therefore are not attenuated during task engagement. The medial prefrontal cortex (mPFC) may encode predictions and prediction errors even when the predicted outcomes are not contingent on prior actions [106,107]. Garofalo et al. [106] revealed that the mediofrontal negativity encodes the unexpected timing of outcomes during a task with no action requirement. The fronto-striatal loops involved in flexible behavioral adaptation could be differentiated in a cortical and subcortical component. Future studies might explore whether these differences can be related to the activation of different brain networks [108]. Furthermore, recent findings have shown that DMN maintains both stability and flexibility naturally and during task-based recognition, and DMN is a causal outflow network in both memory encoding and recall [109]. These findings provide new insights into the electrophysiological foundation of DMN and demonstrate the electrophysiological composition of DMN during cognitive work in a common large network framework. 

QEEG during the resting state can provide information about the balance between large-scale functional brain networks [110]. TGC can play a functional role in multi-scale neuronal communication coordination as a neural correlate that provides information from large-scale functional brain networks to local cortical processing regions [111]. Therefore, our results suggest that resting-state activation and task condition deactivation can be tracked by shifts in TGC at the cortical surface and can reflect the degree of interactions across active neuron clusters of brain network [111,112].

## 5. Limitations and Future Directions

This study has certain limitations, including the wide age range (18–41 years old), and the subjects all being healthy adults, meaning children and adolescent were not assessed. Thus, our results may not be applicable to other populations. Although no significant age effects were observed in the data, future studies with children and adolescent participants remain an important target for research. Since this study investigated neuronal interactions in healthy participants, TGC for clinical symptoms was not evaluated. Future studies in patient groups com-pared to controls are needed to confirm the relationship between TGC and working memory performance in clinical samples. 

Complex measures of WM recruit multiple subprocesses, making it difficult to isolate specific contributions of putatively independent subsystems [113]. As different EEG features are thought to index different WM processes [114,115], we evaluated an independent single memory subprocess using the TGC measured at the maintenance stage to distinguish these functions. In this way, when process-separated VWM tasks are used, EEG features can be evaluated more specifically, and different results can be yielded when evaluating their relationship to WM performance. For example, TGC is proposed to be involved in encoding of WM items. This study remains limited in that its results cannot be generalized to all kinds of memory processes, as it focused solely on the maintenance of VWM. Therefore, future studies are necessary to validate these findings by employing additional single domain WM tasks and to investigate other forms of cross–frequency coupling, not focusing exclusively on gamma and theta bands.

## 6. Conclusions

Few research studies have focused on the relationships between cognitive function and the functional role of resting-state TGC. This study investigated neuronal interaction in healthy participants using an evidence-based, neurophysiological measure of TGC. This is the first study to demonstrate that different analyses of power spectra and TGC evaluate different aspects of cognitive processes. Through posterior fast oscillatory activity nesting into the phase of frontal theta wave, long-range networks can be efficiently either coupled or decoupled, respectively. Based on this mechanism, our results identify neural mechanisms for efficient coordination of competing brain networks in the human brain. Our results provide evidence that TGC may constitute a mechanism for neuronal communication between distant brain regions and frequencies not only during WM maintenance but also during resting-state. The TGC appears to reflect the switching that reflects the balance of resting state and task-related brain networks activity. We suggest that the degree of interactions between active neuron clusters of brain networks can be evaluated through the MI of TGC and resting-state activation and task dependent deactivation can be tracked by shifts in TGC at the cortical surface.

## Figures and Tables

**Table 1:** Demographic and clinical characteristics of healthy volunteers.

Variables	Mean	SD	Min	Max
Age (year)	23.9	4.0	18	41
Sex				
Male, N (%)	31 (55.4)			
Female, N (%)	25 (44.6)			
Handedness, N (%)				
Right, N (%)	53 (94.6)			
Left, N (%)	3 (5.4)			
Education (year)	13.2	1.7	12	16
WURS	20.9	14.3	0	63
LNS	11.8	2.6	7	18
Spatial Span	20.5	3.8	14	29

SD: Standard Deviation, WURS: Wender Utah Rating Scale, LNS: Letter Number Sequencing.

**Table 2:** Comparisons of absolute theta power during the resting state and the retention interval of the VWM task in healthy volunteers.

Lead (μV^2^)	Resting State		VWM Task		t	p_FDR_
	Mean	SD	Mean	SD		
FP2	0.97	0.33	1.06	0.40	−2.07	0.066
Fz	0.99	0.38	1.13	0.44	−3.05 *	0.036
FP1	0.98	0.37	1.07	0.40	−2.01	0.066
F3	0.88	0.32	0.96	0.36	−2.19	0.058
F7	0.84	0.33	0.94	0.37	−2.56 *	0.036
C3	0.60	0.25	0.65	0.26	−1.94	0.072
T3	0.71	0.33	0.82	0.36	−2.70 *	0.036
P3	0.52	0.24	0.57	0.25	−1.69	0.102
T5	0.74	0.36	0.82	0.38	−2.59 *	0.036
Pz	0.55	0.24	0.58	0.23	−1.63	0.108
O1	0.72	0.33	0.79	0.35	−2.90 *	0.036
O2	0.69	0.32	0.76	0.33	−2.74 *	0.036
P4	0.49	0.25	0.55	0.25	−2.18	0.058
T6	0.73	0.40	0.81	0.43	−1.87	0.079
C4	0.56	0.25	0.62	0.26	−2.59 *	0.036
T4	0.67	0.35	0.76	0.38	−2.46 *	0.040
F8	0.81	0.33	0.89	0.40	−2.19	0.058
F4	0.87	0.33	0.96	0.39	−2.03	0.066
Cz	0.71	0.31	0.77	0.33	−1.71	0.102

VWM: Visual Working Memory, SD: Standard Deviation, FDR: False Discovery Rate. * *p* < 0.05.

**Table 3:** Comparisons of absolute gamma power during the resting state and the retention interval of the VWM task in healthy volunteers.

Lead (μV^2^)	Resting State		VWM Task		t	p_FDR_
	Mean	SD	Mean	SD		
FP2	0.21	0.04	0.22	0.06	−1.15	0.286
Fz	0.18	0.04	0.20	0.05	−2.95 *	0.044
FP1	0.21	0.05	0.22	0.06	−2.22	0.083
F3	0.18	0.03	0.19	0.04	−2.15	0.085
F7	0.20	0.05	0.21	0.05	−1.69	0.143
C3	0.13	0.03	0.14	0.03	−0.64	0.523
T3	0.18	0.07	0.20	0.07	−1.81	0.119
P3	0.12	0.03	0.12	0.03	−1.49	0.181
T5	0.16	0.05	0.17	0.05	−1.93	0.102
Pz	0.11	0.03	0.11	0.04	−1.99	0.097
O1	0.17	0.10	0.19	0.12	−1.51	0.181
O2	0.15	0.07	0.18	0.08	−2.70 *	0.046
P4	0.11	0.03	0.12	0.03	−2.66 *	0.046
T6	0.16	0.05	0.17	0.06	−2.08	0.089
C4	0.12	0.03	0.13	0.03	−2.60 *	0.046
T4	0.16	0.05	0.18	0.07	−2.31	0.078
F8	0.19	0.05	0.20	0.05	−1.15	0.286
F4	0.18	0.04	0.18	0.04	−0.69	0.520
Cz	0.12	0.03	0.13	0.04	−3.20 *	0.043

VWM: Visual Working Memory, SD: Standard Deviation, FDR: False Discovery Rate. * *p* < 0.05.

**Table 4:** Comparisons of the MI of TGC during the resting state and the retention interval of the VWM task in healthy volunteers.

Lead (×10^−5^)	Resting State		VWM Task		t	p_FDR_
	Mean	SD	Mean	SD		
FP2	1.31	0.31	1.28	0.29	0.58	0.942
Fz	1.33	0.28	1.30	0.34	0.57	0.942
FP1	1.30	0.31	1.33	0.32	−0.49	0.942
F3	1.27	0.33	1.30	0.28	−0.35	0.946
F7	1.32	0.30	1.31	0.31	0.18	0.946
C3	1.30	0.32	1.41	0.32	−2.07	0.413
T3	1.27	0.34	1.33	0.29	−1.03	0.942
P3	1.33	0.31	1.25	0.32	1.34	0.877
T5	1.32	0.28	1.36	0.34	−0.63	0.942
Pz	1.43	0.78	1.38	0.44	0.46	0.942
O1	1.44	0.45	1.44	0.55	0.04	0.969
O2	1.43	0.57	1.44	0.38	−0.13	0.946
P4	1.37	0.40	1.36	0.44	0.22	0.946
T6	1.40	0.43	1.41	0.37	−0.26	0.946
C4	1.34	0.36	1.23	0.23	1.86	0.434
T4	1.25	0.32	1.31	0.32	−1.01	0.942
F8	1.27	0.32	1.44	0.32	−2.71	0.174
F4	1.33	0.31	1.28	0.31	0.74	0.942
Cz	1.25	0.29	1.28	0.34	−0.53	0.942

MI: Modulation Index, TGC: Theta-Gamma Coupling, VWM: Visual Working Memory, SD: Standard Deviation, FDR: False Discovery Rate.

**Table 5:** Pearson’s correlation coefficients between the absolute gamma power and WURS in healthy volunteers.

Lead	WURS	
	r	p_FDR_
FP2	0.25	0.067
Fz	0.23	0.089
FP1	0.24	0.071
F3	0.18	0.189
F7	0.20	0.133
C3	0.06	0.686
T3	0.03	0.806
P3	−0.06	0.677
T5	−0.10	0.487
Pz	0.24	0.080
O1	0.34 *	0.010
O2	0.46 ^†^	<0.001
P4	0.02	0.901
T6	0.16	0.252
C4	0.15	0.257
T4	−0.09	0.535
F8	0.19	0.168
F4	0.20	0.149
Cz	0.11	0.424

Values are Pearson’s correlation coefficients. * *p* < 0.05, ^†^ *p* < 0.001. WURS: Wender Utah Rating Scale, FDR: False Discovery Rate.

**Table 6:** Linear regression models of WURS as predicted by the absolute gamma power during the VWM task over the occipital scalp sites in healthy volunteers.

	β	B (SE)	t	*p*
Model				0.010
(Constant)		12.46 (13.90)	0.90	0.374
Age	−0.06	−0.21 (0.46)	−0.45	0.657
Sex	−0.03	−0.95 (3.98)	−0.24	0.813
Absolute Gamma Power at O1	0.14	16.38 (19.24)	0.85	0.399
Absolute Gamma Power at O2	0.38	64.46 (24.88)	2.59 *	0.012

Linear regression analyses were performed with the WURS as a dependent variable, and age, sex, and absolute gamma power as independent variables. Standardized regression coefficients (β), Unstandardized coefficients (B) and Standard Errors (SE). * *p* < 0.05. WURS: Wender Utah Rating Scale, VWM: Visual Working Memory.

## Data Availability

The data in this study are available on reasonable request from the corresponding author.
